# Elder Abuse Vulnerability and Risk Factors: Is Financial Abuse Different From Other Subtypes?

**DOI:** 10.1177/07334648211036402

**Published:** 2021-08-07

**Authors:** Silvia Fraga Dominguez, Bee Ozguler, Jennifer E. Storey, Michaela Rogers

**Affiliations:** 1 Glasgow Caledonian University, Glasgow, UK; 2 Royal Holloway, University of London, Egham, UK; 3 University of Kent, Canterbury, UK; 4 The University of Sheffield, Sheffield, UK

**Keywords:** elder mistreatment, material abuse, poly-victimization, management, prevention

## Abstract

Elder abuse (EA) affects one in six older adults, and financial EA, a common subtype, severely impacts victims and society. Understanding victim vulnerability and perpetrator risk factors is essential to EA prevention and management. The limited existing evidence about these factors in relation to EA types suggests that financial EA is different. In a cross-sectional quantitative analysis of secondary data (*N* = 1,238), we investigated EA vulnerability and risk factors, and victim–perpetrator family relationship, with respect to different EA types (financial only, financial co-occurring with other types, and nonfinancial abuse). Financial abuse-only cases had the lowest prevalence of vulnerability and risk factors. Most of these factors, and a familial relationship, were significantly more common in cases involving other EA types. Findings indicate that financial abuse, occurring in isolation, is distinct from other EA types. Risk assessment and future research should consider financial abuse separately to other EA forms.

Elder abuse (EA) (also known as older adult abuse, mistreatment, or maltreatment) can be defined as “a single or repeated act or lack of appropriate action, occurring within a relationship of trust, which causes harm or distress to an older person” (World Health Organization [WHO], 2020, para. 2). EA is a widespread global problem, with a recent study finding a pooled prevalence rate of 15.7% (Yon et al., 2017) and with an estimated prevalence of 4% in the United Kingdom among community-dwelling adults aged 66 and above (O’Keeffe et al., 2007). To prevent and address EA, we need to identify and understand victim vulnerability and perpetrator^1^ risk factors, and use them to guide case management (Storey, 2020). The identification and management of these factors is challenged by the diversity of EA, particularly in terms of the types and abusive behaviors involved.

EA types include financial, physical, psychological, and sexual abuse, and neglect (WHO, 2020). These can be perpetrated in isolation or can co-occur, a dynamic known as poly-victimization (Hamby et al., 2016). Among EA types, the second most common, albeit under-studied, is financial abuse. Financial abuse has a prevalence of 6.8% for adults 60 or older worldwide, and between 0.7% and 1.0% for UK adults aged 66 or older (O’Keeffe et al., 2007; Yon et al., 2017). Financial EA refers to the theft of money and materials, coercion, mismanagement of finances, influence or pressure to resign money or property, and the improper use of legal authority, such as Power of Attorney (Setterlund et al., 2007). Financial EA usually implies actions perpetrated by a trusted individual, differentiating it from fraud or scams, which are perpetrated by strangers (Burnes et al., 2017). Financial EA has severe and lasting impacts: for example, victims can require more health and social care but be unable to pay for it as a direct result of financial loss, a loss which may hinder independent living and quality of life (Age UK, 2015; Jackson & Hafemeister, 2011; Peterson et al., 2014; Rosen et al., 2019). In addition, victims often experience psychologically distressing consequences, including isolation from loved ones, depression, and suicidal thoughts (Deem et al., 2007).

Despite its prevalence and impact, financial abuse is one of the lesser studied forms of EA (Lichtenberg, 2016; Peterson et al., 2014). Nonetheless, available research indicates that there may be specific characteristics in its presentation and unique intervention challenges as a result (Burnett et al., 2020), suggesting the need to respond to and manage it differently than other EA types. Although a debate exists in terms of whether financial abuse is more often premeditated or opportunistic as compared to other EA types, researchers have identified characteristics uncommon in other EA types (e.g., remote perpetration, without victim access) (Setterlund et al., 2007). As such, financial abuse may require the involvement of specific services with specialist expertise and, thus, may not be tackled effectively with a general EA management approach. For example, legal intervention within the criminal justice system may be more successful compared to a safeguarding approach involving social services (Brownell & Wolden, 2003). In addition, assessing capacity related to financial decision-making and management while involving professionals from financial services could be more effective in financial abuse cases (Lichtenberg, 2016).

Notwithstanding the diversity of abuse types, abusive behaviors, and the suggestion that financial abuse may be a distinct type, EA has been traditionally studied as a whole (Jackson & Hafemeister, 2011). This one-size-fits-all approach is criticized by Jackson and Hafemeister (2011), who attribute some of the inconsistency of findings in the field of EA to the lack of acknowledgment of different dynamics and risk factors depending on abuse type. Although there have been some efforts to study each abuse type separately and, within that, evidence that financial abuse may diverge from other types (Burnett et al., 2020), empirical evidence for risk factors of specific subtypes of EA has been limited (Jackson & Hafemeister, 2011; Johannesen & LoGiudice, 2013).

Studying each abuse type separately does have limitations, however, as evidence suggests that older adults often experience poly-victimization, that is, more than one type of abuse simultaneously (Hamby et al., 2016; Williams et al., 2020). Thus, victims of financial abuse are also frequently victimized in other ways, and research by Jackson and Hafemeister (2014) found unique risk factors (i.e., codependency) associated with financial abuse co-occurring with other types. This research studied financial abuse co-occurring with physical abuse and/or neglect but did not examine it in relation to other types of abuse (e.g., psychological, the most prevalent EA type; Yon et al., 2017). Research has rarely addressed poly-victimization, so there is limited updated knowledge about these potential differences.

EA is also diverse in terms of relationship patterns, although most EA perpetrators are related to their victims, frequently as their adult children or partners (Deliema et al., 2018; Jackson & Hafemeister, 2014). There is research supporting differences in terms of the types of EA perpetrated, based on the type of relationship between victim and perpetrator. Jackson and Hafemeister (2011) compared relative perpetrators (child, grandchild, spouse, and other relative) to nonrelative perpetrators (professional caretaker, friend, neighbor, and stranger) and found that, while financial abuse was more likely to be perpetrated by nonrelatives, financial abuse co-occurring with neglect or physical abuse was more likely to be perpetrated by relatives. More recently, Weissberger and colleagues (2020) similarly found that family members were more likely to engage in poly-victimization as compared to nonrelative perpetrators.

Important areas to foreground within a discussion of EA prevention and intervention are victim vulnerability and perpetrator risk factors, particularly those that are dynamic (i.e., those that can be changed through intervention). Victim vulnerability factors are those that place an older adult at greater risk of abuse, and perpetrator risk factors those that increase the risk of an individual perpetrating abuse (Storey, 2020). A consideration of victim vulnerability and perpetrator risk factors is consistent with the Risk Need Responsivity model; an influential model in offender assessment and treatment in other fields that has guided the development of effective risk assessment tools that embed dynamic risk factors (Bonta & Andrews, 2007). Well-supported dynamic victim vulnerability and perpetrator risk factors for EA were recently identified in a literature review by Storey (2020) and underpin this work. These factors have not been frequently studied in the context of abuse type or co-occurrence of different subtypes (Jackson & Hafemeister, 2011). However, several findings indicate that risk factors may be more relevant in certain abuse subtypes (Jackson, 2016). To develop effective ways of identifying and managing cases of financial EA, it must be understood whether certain risk factors (e.g., financial dependency) are more likely to be present in financial EA only cases as opposed to other EA types or cases where financial abuse is perpetrated along with other abuse types (i.e., poly-victimization). Furthering this understanding will help in advancing research on EA risk assessment and management.

This study investigated EA by comparing cases involving financial abuse only, financial abuse that co-occurs with other abuse types (i.e., physical, psychological, sexual, and neglect), and EA that is not financial in nature. Specifically, this study aimed to understand whether these abuse types were characterized by a different:

1. Frequency of victim vulnerability and perpetrator risk factors;
2. Nature of victim vulnerability and perpetrator risk factors;
3. Victim–perpetrator relationship (family vs nonfamily).

## Design and Methodology

### Research Design and Data Source

This study involved quantitative analyses of cross-sectional data from Age UK, a major national charity in the United Kingdom that provides services, including information and advice, to older people. One of their services is a national helpline, which the public can contact via phone or email for information and advice regarding themselves or others. The details of these enquiries are recorded in case notes by staff. Cases that raise concerns about or allege the presence of EA are flagged as such. The researchers were granted access to all the case notes flagged as EA between April 2014 and March 2017 (*N* = 1,411). Cases were fully anonymized prior to providing researchers access to ensure confidentiality. An information sharing agreement was signed by all parties, and ethical approval was gained from the first author’s university Ethics Committee. The data source and coding process are further described in Table 1.

**Table 1.** Timeline and Coding Process.

Area	Details
Data source	The data were anonymized cases obtained from the charity Age UK’s helpline that were previously flagged as EA cases by the helpline staff over a period of 3 years (April 2014–March 2017). Each case included a description of the concerns reported by the enquirer and recorded by Age UK staff. This case description generally encompassed details about the individuals involved in the case (i.e., alleged victim and perpetrator), their characteristics, the victim–perpetrator relationship, and the abusive situation (e.g., a description of the type of abuse and abusive behaviors).
Timeline for coding	February 2018–July 2019
Steps followed	1. Two of the authors developed a data collection tool to gather information on the variables of interest (described in “Materials”) prior to the start of the coding process. These variables related to four main areas: sample characteristics, relationship between the victim and perpetrator, vulnerability and risk factors, and abuse type(s). The procedure of extracting case characteristics from secondary data has been employed previously by researchers in the field (e.g., Storey & Perka, 2018; Weissberger et al., 2020). 2. Two coders independently assessed and extracted the relevant data from the cases. Coding involved carefully reading the cases, extracting the information using the data collection tool, and entering the data into SPSS in preparation for data analysis. a. First, the two coders coded several practice cases together to ensure consistency and resolve disagreement. b. Second, and once consistency was reached in ‘a’, the next 86 cases (7%) were coded by both coders separately to calculate inter-rater reliability early in the process. As inter-rater reliability was high, a decision was made not to double code and compare further cases. All further data were coded by one of the researchers.

### Data and Variables

A coding scheme was developed to gather the data needed for the purposes of this study. The coding scheme recorded primarily nominal variables, some of them with categories (e.g., victim’s relationship with the perpetrator) but most coded dichotomously as present or absent (e.g., physical health problems). The following variables were recorded:

#### Sample characteristics

*Demographics*: gender and age of the victim and perpetrator. *Reporter identity*: identity of the person who reported the EA to Age UK: victim or nonvictim (and relationship to the victim).

#### Relationship between the victim and perpetrator

Relationships were coded as family or nonfamily and entered in the logistic regression analyses. Family relationships included perpetrators who were the adult child, partner or ex-partner, grandchild, niece/nephew, sibling, parent, or other family member of the victim. Nonfamily perpetrators included neighbor, friend or acquaintance, stranger, professional, and other nonfamily member. Although the WHO definition excludes strangers, they were retained in the sample because Age UK considered them as EA cases.

#### Vulnerability and risk factors

The victim vulnerability and perpetrator risk factors coded as present or absent herein were identified from a literature review encompassing 198 studies in the field of EA (Storey, 2020) and are summarized in Table 2. For both the vulnerability and risk factors, a numerical continuous variable was created adding all the present factors (ranging from 0 to 7 for vulnerability factors and from 0 to 6 for risk factors). This total number was studied to understand whether a higher number of vulnerability/risk factors was associated to a specific EA type, as this could indicate higher risk and the need for more intensive intervention (Bonta & Andrews, 2007; Hart et al., 2016).

**Table 2.** Vulnerability and Risk Factors Based on a Systematic Review (Storey, 2020).

Name	Definition	
	Victims	Perpetrators
Physical health problems^a^	Poor physical health, medical problems, or physical disability (Beach et al., 2005; Dong, 2015; Eisikovits et al., 2004; Johannesen & LoGiudice, 2013; Lachs & Pillemer, 2015).	
Mental health problems^a^	Diminished psychological health, cognitive impairment (including the diagnosis of dementia), intellectual disability, the recent worsening of cognitive abilities, and mental health problems, such as depression and anxiety (Acierno et al., 2010; Dong et al., 2014; Jackson, 2016; Johannesen & LoGiudice, 2013; Lachs et al., 1997; Wiglesworth et al., 2010).	
Dependency	Care or financial dependency (Jackson & Hafemeister, 2014; Pillemer et al., 2007).	Financial dependency (Jackson & Hafemeister, 2011).
Problematic attitudes	Wanting to protect the abuser by minimizing the abuse or displaying ambivalent or inconsistent opinions regarding the perpetrator’s behaviors (Henderson et al., 2002; Jackson & Hafemeister, 2014).	Ageism, antisocial behavior such as hostility, a history of criminal behavior, and unrealistic expectations of the victim (Anme et al., 2006; Johannesen & LoGiudice, 2013; Lachs & Pillemer, 2015; Olofsson et al., 2012).
Previous victimization	Previous abuse experienced or witnessed, other than the current episode of EA (Fulmer et al., 2005; Johannesen & LoGiudice, 2013).	Previous abuse experienced or witnessed during the perpetrator’s childhood or adolescence (Jackson & Hafemeister, 2011).
Substance abuse problems^a^	Problems related to the use of illegal substances or misuse of legal substances, such as alcohol (Anetzberger et al., 1994; Henderson et al., 2002; Jackson, 2016; Kosberg, 1988).	
Cohabitation^b^	Cohabitation with the perpetrator (Jackson & Hafemeister, 2011; Johannesen & LoGiudice, 2013; Peterson et al., 2014).	

*Note.* EA = elder abuse.

a The definition for victims and perpetrators is the same. ^b^This factor is only examined for victims.

#### Abuse type(s)

The dependent/outcome variable for the purpose of analysis was the abuse type(s) described in the case. Abuse types included the presence or absence of financial, physical, psychological, or sexual abuse and/or neglect. For the purpose of data analysis, this variable was re-categorized into (a) financial abuse only (FAonly), defined as cases where financial abuse was coded as present and all the other types were coded as absent; (b) financial abuse co-occurring with other abuse types (FAco-occurring), defined as cases where financial abuse was coded as present and there was at least one other EA type coded as present; and (c) nonfinancial abuse (non-FA), defined as cases where financial abuse was coded as absent and one or more of the other EA types was coded as present.

### Exclusion Criteria

Some cases (*n* = 173, 12.3%) lacked adequate detail to code the relevant factors in this study and thus were excluded from analysis. Cases were excluded if (a) they lacked information about both abuse type and victim–perpetrator relationship; (b) the reporter asked general questions about EA but gave no details regarding an instance of abuse; or (c) the cases involved self-neglect, because these would not include a separate victim and perpetrator for whom to code vulnerability and risk factors.

### Inter-Rater Reliability

Two raters were involved in data coding (S.F.D. and B.O.). To assess reliability, a subset of the data was coded by both authors. Inter-rater reliability was calculated for these cases using Cohen’s Kappa for categorical variables and Intraclass Correlation Coefficient (ICC; two-way, mixed methods, absolute agreement) for continuous variables (i.e., age and number of risk factors). Results are reported in Table 3. Cohen’s Kappa results ranged from .79 to 1, indicating good to very good agreement (Altman, 1999), and ICC_1_ ranged from .96 to 1, indicating excellent agreement (Koo & Li, 2016).

**Table 3.** Inter-Rater Reliability Results.

Variable		Kappa	ICC	Percent agreement (%)
Type of abuse		1		
Type of relationship		1		
Enquirer identity		1		
			
Victim variables	Gender	1		
	Age		1	
	Number of vulnerability factors		.99	
	Mental health problems	1		
	Physical health problems	1		
	Dependency	.96		
	Problematic attitudes	1		
	Previous victimization	.88		
	Substance abuse			100
	Living together	1		
Perpetrator variables	Gender	1		
	Age		1	
	Number of risk factors		.96	
	Mental health problems	1		
	Physical health problems	.79		
	Financial dependency	.91		
	Problematic attitudes	.95		
	Previous victimization			100
	Substance abuse			100

*Note.* Percent agreement was calculated when Cohen’s Kappa could not be calculated because the variable was a constant.

### Data Analysis

This study employed a Kruskal Wallis H to test whether there was a different number of vulnerability or risk factors for different abuse types, for the victim and perpetrator, respectively. This test was used instead of a one-way analysis of variance (ANOVA) because the data were not normally distributed and there were outliers. To investigate if different factors were associated with abuse type, two multinomial regressions were employed, one with victim vulnerability factors and one with perpetrator risk factors. In both models, the victim–perpetrator relationship was also considered. Thus, the vulnerability/risk factors and the victim–perpetrator relationship were entered as explanatory variables, with the abuse type as the outcome. SPSS version 21 was used for the analyses.

## Results

A total of 1,238 cases met inclusion criteria and were fully coded (87.7% of the cases recorded across the period; *N* = 1,411). Victims’ ages ranged from 53 to 104 (*M* = 83.9, *SD* = 8.8) and they were predominantly female (*n* = 805, 65%; 1.6% missing data). Age UK has no age cut-off for the use of the helpline; however, only three cases concerned victims younger than 60. These three cases were included because the helpline flagged them as EA. Perpetrators’ ages ranged from 9 to 96 (*M* = 55.5, *SD* = 21.9) and they were more often male (*n* = 519, 41.9%; 10.6% missing data). Many cases involved FAonly (*n* = 585, 47.4%), followed by FAco-occurring (*n* = 347, 28.1%), and non-FA (*n* = 302, 24.5%). The frequencies of specific EA types can be found in Table 4. Victims were self-reporting in a minority of cases (*n* = 138, 11.1%), with cases primarily reported by a family member (*n* = 897, 72.5%). Adult children were the most common perpetrators, followed by a partner/ex-partner (see Table 5).

**Table 4.** Prevalence of Abuse Type.

Abuse type		*N*	%
Financial abuse only		585	47.4
Financial abuse co-occurring		347	28.1
	Co-occurring with psychological	236	19.1
	Co-occurring with neglect	51	4.1
	Co-occurring with other EA type	60	4.9
Nonfinancial abuse		302	24.5
	Physical	26	2.1
	Psychological	196	15.9
	Neglect	10	0.8
	Sexual	4	0.3
	Nonfinancial poly-victimization	66	5.4

*Note.* Four cases missing. Financial abuse cases (only or co-occurring) most commonly involved the following behaviors: control of finances (19%), stealing money, valuables, or both (17%), taking money with the victim’s consent (15%), abusing their Power of Attorney (15%), or a combination of behaviors (11%). EA = elder abuse.

**Table 5.** Victim–Perpetrator Relationship by Abuse Type.

Abuse type	Victim–perpetrator relationship	*N*	%
Financial abuse only			
	Family	395	32.5
	Adult child (including adopted and step)	280	23.0
	Partner	34	2.8
	Ex-partner	1	0.1
	Grandchild	19	1.6
	Niece/nephew	19	1.6
	Sibling	5	0.4
	Other family member	25	2.1
	Multiple family members	12	1.0
	Nonfamily	178	14.6
	Professional (including carers and legal professionals)	73	6.0
	Friend or acquaintance	66	5.4
	Neighbor	16	1.3
	Stranger	11	0.9
	Other nonfamily	12	1.0
Financial abuse co-occurring			
	Family	298	24.5
	Adult child (including adopted and step)	206	16.9
	Partner	35	2.9
	Ex-partner	5	0.4
	Grandchild	17	1.4
	Niece/nephew	11	0.9
	Sibling	8	0.7
	Other family member	8	0.7
	Multiple family members	8	0.7
	Nonfamily	46	3.8
	Professional (including carers and legal professionals)	16	1.3
	Friend or acquaintance	20	1.6
	Neighbor	7	0.6
	Other nonfamily	3	0.3
Nonfinancial abuse			
	Family	264	21.7
	Adult child (including adopted and step)	134	11.0
	Partner	78	6.4
	Ex-partner	4	0.3
	Grandchild	10	0.8
	Niece/nephew	7	0.6
	Sibling	9	0.7
	Parent	10	0.8
	Other family member	10	0.8
	Multiple family members	2	0.2
	Nonfamily	35	2.9
	Professional (including carers and legal professionals)	14	1.1
	Friend or acquaintance	9	0.7
	Neighbor	3	0.3
	Stranger	1	0.1
	Other nonfamily	8	0.7

*Note.* 22 cases missing. Total family: 957 (78.7%); total nonfamily: 259 (21.3%).

### Comparisons in Number of Vulnerability and Risk Factors by Abuse Type

#### Number of victim vulnerability factors

A Kruskal–Wallis *H*-test was conducted to determine if there were differences in the number of vulnerability factors between the three EA types. The distributions of the number of vulnerability factors were dissimilar across groups, as assessed by visual inspection of the boxplot; thus, mean ranks are compared instead of medians. There were significant differences in the mean ranks of the number of vulnerability factors between the three EA types, χ^2^ (2) = 44.651, *p* < .001. Pairwise comparisons were conducted using Dunn’s (1964) procedure with a Bonferroni correction for multiple comparisons. Adjusted *p* values are presented. There was a significant difference in the number of vulnerability factors across abuse types, where non-FA (mean rank = 678.80) cases had significantly more vulnerability factors than FAonly (mean rank = 541.68) cases (*p* < .001) and FAco-occurring (mean rank = 662.52) cases had significantly more vulnerability factors than FAonly (*p* < .001) cases. There was no difference in the number of vulnerability factors between FAco-occurring and non-FA (*p* = 1.00).

#### Number of perpetrator risk factors

A Kruskal–Wallis *H*-test was also conducted to determine if there were differences in the number of risk factors between the three EA types. The distributions of the number of risks factors were dissimilar across groups, as assessed by visual inspection of the boxplot; thus, mean ranks are compared. There were significant differences in the mean ranks of the number of risk factors between the three EA types, χ^2^ (2) = 164.961, *p* < .001. Pairwise comparisons were conducted. Non-FA cases had significantly more risk factors (mean rank = 766.05) than FAonly (mean rank = 500.40) cases (*p* < .001) and FAco-occurring (mean rank = 681.58) (*p* = .002) cases. Finally, FAco-occurring cases had significantly more risk factors than FAonly (*p* < .001).

### Comparisons of Vulnerability and Risk Factors by Abuse Type

Multinomial logistic regression was used to assess whether different victim vulnerability and perpetrator risk factors and the victim–perpetrator relationship contributed to variances in abuse types.

#### Victim vulnerability factors and abuse type

A model with victim vulnerability factors and victim–perpetrator relationship as explanatory variables was statistically significant and a good fit of the data (Table 6). Of the eight predictors, four were statistically significant: victim’s mental health problems, victim’s dependency, co-habitation, and family relationship. Victims with mental health problems were more likely to be suffering FAonly as compared to both FAco-occurring and non-FA (odds ratio [OR] = 1.56, OR = 1.72; respectively). The victim’s dependency, co-habitation, and family relationship with the perpetrator were all significant predictors of FAco-occurring and non-FA as compared to FAonly. Victims who were living with the perpetrator were 6.18 times more likely to be suffering non-FA and 2.57 times more likely to be suffering FAco-occurring as compared to FAonly. In addition, previous victimization explained non-FA as compared to FAonly.

**Table 6.** Model of Victim Vulnerability Factors and Abuse Type.

Variables	*B* (*SE*)	95% CI for odds ratio		
		Lower	Odds ratio	Upper
**FA co-occurring vs FAonly**				
Intercept	−1.88 (.21)***			
Physical health problems	0.21 (.17)	0.88	1.23	1.72
Mental health problems	−0.45 (.16)**	0.46	0.64	0.88
Dependency	0.97 (.17)***	1.89	2.64	3.68
Problematic attitudes	−0.04 (.21)	0.64	0.96	1.44
Previous victimization	0.39 (.36)	0.73	1.48	3.00
Substance abuse	0.35 (.62)	0.42	1.42	4.80
Living together	0.94 (.17)***	1.85	2.57	3.56
Family relationship	1.16 (.20)***	2.17	3.19	4.70
**non-FA vs FAonly**				
Intercept	−2.12 (.23)***			
Physical health problems	0.15 (.18)	0.81	1.16	1.66
Mental health problems	−0.54 (.18)**	0.41	0.58	0.83
Dependency	0.46 (.19)*	1.09	1.58	2.29
Problematic attitudes	−0.43 (.24)	0.41	0.65	1.04
Previous victimization	0.84 (.36)*	1.14	2.32	4.72
Substance abuse	0.08 (.71)	0.27	1.09	4.32
Living together	1.82 (.17)***	4.42	6.18	8.63
Family relationship	1.07 (.22)***	1.88	2.90	4.48

*Note.* Omnibus test of model coefficients: χ^2^(16, *N* = 1,200) = 266.691, *p* < .001; Model fit (Hosmer and Lemeshow Test): χ^2^(174, *N* = 1,200) = 175.868, *p* = .446; Nagelkerke = .227. Cases correctly classified: 56.2%. CI = confidence interval; FA = financial abuse.

* *p* < .05. **p < .01. ***p < .001.

#### Perpetrator risk factors and abuse type

A model with perpetrator risk factors and victim–perpetrator relationship as explanatory variables was significant, but it was not a good fit of the data (see Table 7). The predictor “previous victimization” was eliminated from the model because this variable was a constant. Of the remaining six predictors, five of them were statistically significant: physical health problems, mental health problems, problematic attitudes, substance abuse, and victim–perpetrator relationship. Mental health problems, problematic attitudes, and a family relationship with the victim were significant predictors of FAco-occurring and non-FA as compared to FAonly. Perpetrators with mental health problems were 10.36 times more likely to perpetrate non-FA and 3.53 times more likely to perpetrate FAco-occurring, both as compared to FAonly. In addition, physical health problems (OR = 5.23) and substance abuse (OR = 2.76) were significant predictors of non-FA as compared to FAonly.

**Table 7.** Model of Perpetrator Risk Factors and Abuse Type.

Variables	*B* (*SE*)	95% CI for odds ratio		
		Lower	Odds ratio	Upper
FA co-occurring vs FAonly				
Intercept	−1.68 (.18)***			
Physical health problems	0.36 (.55)	0.48	1.43	4.24
Mental health problems	1.26 (.39)**	1.63	3.53	7.67
Financial dependency	−0.06 (.45)	0.39	0.94	2.29
Problematic attitudes	1.22 (.16)***	2.49	3.39	4.61
Substance abuse	0.39 (.39)	0.68	1.48	3.19
Family relationship	0.95 (.19)***	1.79	2.58	3.73
non-FA vs FAonly				
Intercept	−2.05 (.20)***			
Physical health problems	1.66 (.47)***	2.07	5.23	13.21
Mental health problems	2.34 (.37)***	5.04	10.36	21.31
Financial dependency	−0.95 (.55)	0.13	0.39	1.14
Problematic attitudes	1.21 (.17)***	2.41	3.36	4.69
Substance abuse	1.02 (.38)**	1.32	2.76	5.78
Family relationship	0.98 (.21)***	1.76	2.68	4.06

*Note*. Omnibus test of model coefficients: χ^2^(12, *N* = 1,213) = 236.867, *p* < .001; Model fit (Hosmer and Lemeshow Test): χ^2^(58, *N* = 1,213) = 104.500, *p* < . 001; Nagelkerke = .171. Cases correctly classified: 55.5%. CI = confidence interval; FA = financial abuse.

* *p* < .05. **p < .01. *** *p* < .001.

## Discussion

The purpose of our study was to understand whether there were differences in the number and type of vulnerability and risk factors, and victim–perpetrator relationships, associated with different types of EA victimization or perpetration. Specifically, this study investigated EA involving financial abuse only, financial abuse that co-occurs with other abuse types, and nonfinancial EA. Results showed significant differences in the number and type of vulnerability and risk factors associated with different abuse types. Specifically, there were more vulnerability and risk factors in cases of non-FA and FAco-occurring when compared with FAonly. It was also found that the victim–perpetrator relationship (familial vs nonfamilial) was significantly related to EA type when considered along victim vulnerability factors and perpetrator risk factors. The results support the need for further research to examine financial abuse separately to other forms and identify differences, which will help to provide more specific and effective responses to EA.

In this study, there were fewer vulnerability and risk factors associated with financial abuse compared to other types of EA. This finding has several possible implications. First, it suggests that older victims of financial abuse either have fewer or different vulnerability factors to those commonly reported in the literature (e.g., those reviewed by Storey, 2020). This indicates the need for further study and for modifying risk assessment based on abuse type. Second, the reduced number of risk factors may reflect different perpetrator motivation by abuse type. For instance, financial abuse may have clearer or more singular motives (e.g., financial gain) than other abuse types, such as physical or psychological abuse, where motives may be multiple and complex (e.g., a lack of personal responsibility or concern for others) and stem from the presence of more risk factors (e.g., comorbid mental health and substance abuse problems and problematic attitudes; Storey, 2020). Third, the results may reflect that cases involving more severe abuse as well as more types of offending (e.g., physical, poly-victimization, respectively) are the product of an increased number of vulnerability and risk factors which is in line with previous work and theory (i.e., the Risk Needs Responsivity model where increased needs—or risk factors—are associated with and increased risk for violence; Bonta & Andrews, 2007). For instance, increased vulnerability (e.g., dependency and mental health problems) may result in abuse that is hidden, allowing it to escalate and proliferate over time (Eisikovits et al., 2004; Henderson et al., 2002) and increased risk factors could destabilize and disinhibit the perpetrator, resulting in more severe abuse (Hart et al., 2016).

In terms of the specific vulnerability factors, in cases of non-FA, victims were more likely to be living with the perpetrator, have experienced previous victimization, and be dependent on the perpetrators financially or for care. Co-habitation and dependency were also more common risk factors in FAco-occurring when compared to FAonly. Victim dependency has consistently been identified as a vulnerability factor for EA (Lachs & Pillemer, 2004; Olofsson et al., 2012). Victim dependency can decrease help-seeking behaviors and increase isolation of the victim, making it easier for perpetrators to engage in multiple forms of EA—including financial abuse. Co-habitation could have a similar effect by facilitating the perpetrator’s isolation of and access to the victim and hindering or delaying help-seeking (Fraga Dominguez et al., 2021).

In previous research, studies have consistently found mental health problems and/or cognitive problems to be a vulnerability factor in EA victimhood (Acierno et al., 2010; Dong et al., 2014; Jackson, 2016; Johannesen & LoGiudice, 2013). Our findings support the view that mental health or cognitive problems can create barriers to identifying and comprehending financial abuse (Jackson & Hafemeister, 2011). In addition, these vulnerabilities may lead to the loss of power over personal finances and allow a perpetrator to gain financial control. Although further understanding of how mental health problems and cognitive problems independently contribute to vulnerability to financial abuse is needed, the overall increased vulnerability should shape preventive intervention strategies. For example, at the time of diagnosing an older adult with a mental health (e.g., depression) or cognitive problem, preventive safeguarding action could be undertaken by providing information to the older person and their family about financial decision-making. Furthermore, training and involving frontline financial services may be helpful in addressing risk linked to cognitive impairment or psychological vulnerability (Lichtenberg, 2016).

Non-FA perpetrators were more likely to have mental or physical health problems, substance abuse problems, and problematic attitudes, when compared to FAonly. Mental health problems and problematic attitudes were also more common in FAco-occurring when compared to FAonly. Thus, the presence of mental health problems and/or cognitive problems is a more common risk factor for the perpetration of abuse that is not exclusively financial. This could be due to various reasons. For example, cognitive impairments associated with dementia (Beach et al., 2005; Miller et al., 2006) may make it harder for perpetrators with these impairments to engage in common financial abuse behaviors that require a degree of planning, such as controlling the victim’s finances or misusing a Power of Attorney (Setterlund et al., 2007). Mental health conditions such as depression or bipolar disorder can decrease emotional and impulse control, making reactive forms of EA, like physical abuse, more likely than planned forms of abuse such as financial abuse (which is not to say that financial abuse cannot be reactive rather than planned) (Henderson et al., 2002). As a risk factor, problematic attitudes have sometimes been linked with the existence of a caregiving relationship; for example, when discussing the presence of unrealistic expectations about what the victim can do. Thus, it could be associated to cases of neglect, psychological, or physical abuse (Liu et al., 2019; Storey, 2020).

Finally, this study’s findings are consistent with previous research that has found that victim–perpetrator relationship is associated with abuse type. In this case, family members were more likely to perpetrate EA that was not financial or financial abuse co-occurring with other abuse types. An explanation for this finding is that family members’ usual closeness to the victim as compared to other perpetrators may make it easier to perpetrate different abuse types for which access to the victim is necessary (e.g., physical). The association between a family relationship and the perpetration of poly-victimization is consistent with previous findings by Jackson and Hafemeister (2011) and Weissberger et al. (2020) and stresses the importance of considering victim–perpetrator relationship in research and interventions.

### Limitations

This study is limited by the use of secondary data extracted from helpline records. For example, some relevant variables could have been omitted from case notes due to time constraints or selective reporting. The use of these data may carry concerns related to the measurement of variables; nevertheless the inter-rater reliability results provide confidence about the reliability of coding from the available free texts. Another limitation is that temporality cannot be established and that some of the risk and vulnerability factors analyzed could also be consequences of the abuse. However, even if this were the case, both vulnerability and risk would still need to be managed to reduce the possibility of continued abuse. Despite these limitations, the benefits of analyzing enquiries made to a national helpline have been recently highlighted (Weissberger et al., 2020). The data we analyzed represent the type and amount of information available to helpline staff who make decisions, therefore our findings and recommendations are directly relevant to frontline services. Finally, the multinomial logistic regression findings for the perpetrator model should be interpreted with caution, given that, even though the model was significant, it was not a good fit of the data.

### Implications for Practice and Policy

Our findings indicate that some of the vulnerability and risk factors may not be the best explanatory variables for the victimization or perpetration of financial abuse that occurs in isolation. However, they identify a range of vulnerability and risk factors commonly recognized in EA cases in general. The results do suggest that cases of financial abuse only are distinct in terms of both victim vulnerability and perpetrator risk factors; thus, caution is needed when considering blanket intervention and policy for EA cases. The findings reinforce the need for separate handling of financial EA cases and other EA cases by people working in sectors such as health, social care, and, where relevant, criminal justice. Findings also support the use of EA risk assessment instruments that distinguish between financial abuse only and other forms of EA, such as the *Harm to Older Persons Evaluation* or HOPE (formerly the EARLI; Storey et al., 2020).

Considering the findings for financial abuse only, identifying the impact of mental health or cognitive problems in victims could be relevant in these cases. At the point of an older person’s diagnosis, there needs to be a preventive and safeguarding approach, by making links with family, social care, and legal institutions to increase protection of the person’s assets (Lichtenberg et al., 2019).

The findings highlight the need to identify poly-victimization, given that cases involving FAco-occurring with other types were distinct from cases involving FA only and more similar to non-FA. Thus, when practitioners encounter financial abuse they should screen for, and assess if relevant, other EA types. This is particularly salient in the case of familial relationships, as familial perpetrators in this sample were more likely to engage in FAco-occurring than FAonly.

## Notes

1. For clarity, those alleged to have engaged in EA will be referred to as perpetrators and those who were subjected to abuse as victims.
